# Medial Clavicular Osteophyte: A Novel Cause of Paget-Schroetter Syndrome

**DOI:** 10.1155/2015/723182

**Published:** 2015-04-16

**Authors:** Keagan Werner-Gibbings, Steven Dubenec

**Affiliations:** Department of Vascular Surgery, Royal Prince Alfred Hospital, Sydney, NSW 2006, Australia

## Abstract

Paget-Schroetter syndrome is a form of upper limb deep venous thrombosis usually seen in younger patients in association with repetitive activities of the affected limb. When occurring in more elderly patients or in those where it is difficult to appreciate a causative mechanism, other aetiologies should be considered. We present a case in which degenerative osteoarthritis of the sternoclavicular joint with osteophyte development impinged on the subclavian vein, leading to extensive upper limb thrombosis. The difficulties in identifying and managing this unusual cause of Paget-Schroetter are presented and discussed.

## 1. Introduction

Paget-Schroetter Syndrome is a deep venous thrombosis (DVT) of the subclavian-axillary vein complex usually seen in association with repetitive upper limb activity [1]. Anatomical variations that act to narrow the thoracic outlet such as cervical ribs and hypertrophied musculature are known to predispose towards the development of this condition. Less common causative mechanisms such as posterior dislocation of the clavicular heads and Langer's axillary arch have also been reported. We report a unique case of the investigation and management of Paget-Schroetter Syndrome caused by a large clavicular head osteophyte.

## 2. Case Report

A 69-year-old former competitive rower and active sportsman presented to the emergency department with a 10-day history of pain and increasing swelling to his left arm. His background history included hypertension and paroxysmal atrial fibrillation. Physical examination revealed a neurovascularly intact left arm with extensive swelling distal to the axilla. No other abnormality was detected on physical examination. The patient was left-handed. Venous duplex ultrasound demonstrated occlusive thrombus in the left upper limb venous system involving the proximal subclavian, axillary and basilic veins, and the brachial veins to the level of midhumerus. Laboratory testing showed no evidence of a coagulation disorder.

**Figure 1:**
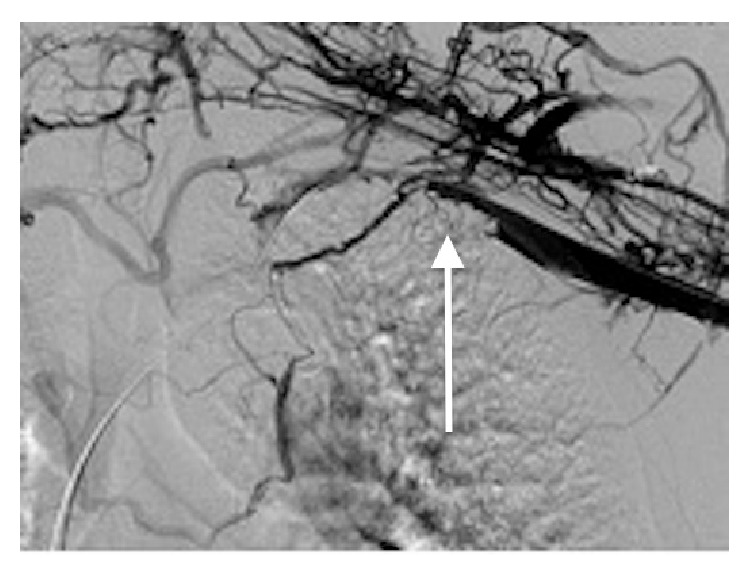
Venogram of Left subclavian vein demonstrating thrombus extending into the left brachiocephalic vein.

Contrast venography confirmed thrombus extending from the brachial vein to the left brachiocephalic vein (Figure 1). Mechanical thrombectomy (Angiojet, MEDRAD Inc.) was employed to decrease thrombotic burden in the affected vessels. Subsequently an infusion catheter was placed in the left brachiocephalic vein. A urokinase infusion was initiated through with daily venography demonstrating resolution of thrombus distal to the first rib following 48 hours of treatment. Angioplasty of the subclavian-axillary system with a 12 mm balloon demonstrated impingement at the level of the first rib. CT angiography of the thoracic outlet showed poor filling of the left upper limb venous system with focal narrowing of the left subclavian artery as it passed over the first rib, suspecting the presence of a fibromuscular band of the first rib causing subclavian vein compression.

Following thrombolysis, the patient underwent a left-sided transaxillary first rib resection to decompress the thoracic outlet with the aim of subsequently placing a stent across the affected portion of subclavian vein. During stent placement, performed 10 days after his first rib resection, the patient's left-sided central veins had again thrombosed, necessitating further mechanical thrombectomy to restore patency. A venous stent was then placed at the level of venous stenosis with poststenting venography, demonstrating a widely patent vessel. The patient was discharged on oral anticoagulation therapy.

**Figure 2:**
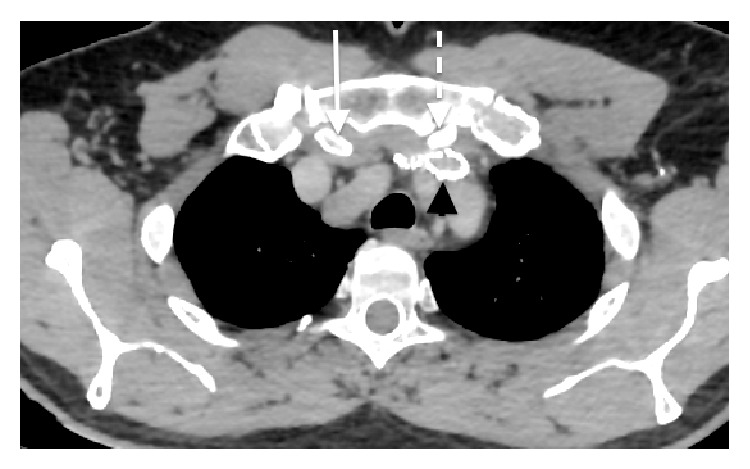
Axial view of large osteophytic projection arising from the posterior surface of the left clavicular head impinging the left subclavian stent.

**Figure 3:**
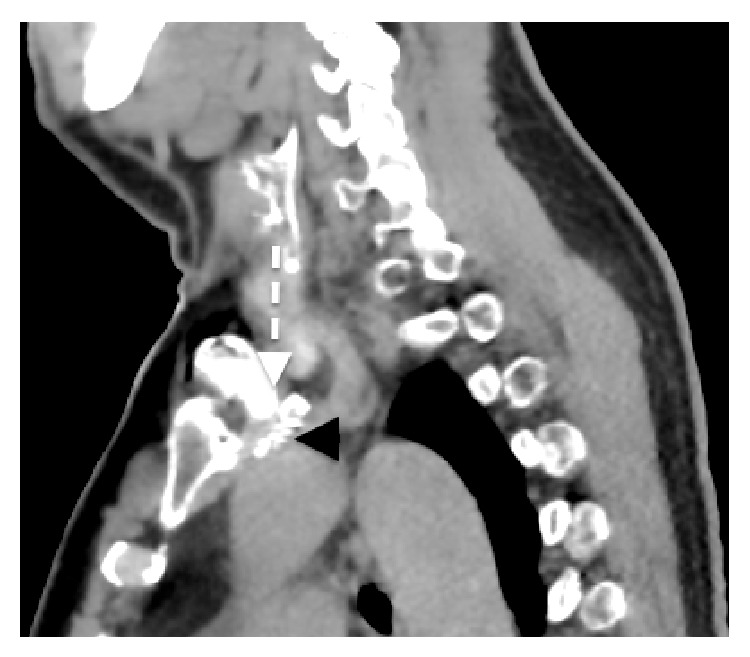
Sagittal view of left subclavian stent impingement.

The patient was symptom-free at his 3-month follow-up; however, a CT at that time demonstrated compression of the subclavian vein stent with significant architectural distortion. The causative mechanism of this impingement was a large osteophytic projection arising from the posterior surface of the left clavicular head (Figures 2 and 3). Dedicated imaging of the sternoclavicular joints bilaterally exposed further signs of severe arthrosis and posterosuperior joint subluxation, indicating advanced osteoarthritic degenerative disease of the joint. The significance of the compression exerted by this large projection was not appreciated on the initial imaging and was likely the initiating factor of the primary thrombotic event.

A specialist shoulder surgeon was consulted on the possibility of surgical resection of the osteophytic prominence to relieve the obstruction. The expert orthopaedic opinion was that resection of the entire medial segment of clavicle was the only available course of treatment. This procedure would necessarily result in a significant functional deficit for the patient where previously none existed. The decision was therefore made to treat medically with anticoagulation and ongoing review. The patient remains symptom-free 6 months after the initial event.

## 3. Discussion

Effort thrombosis or Paget-Schroetter Syndrome is a form of upper limb DVT that occurs when strenuous activity of the upper limb results in subclavian vein endothelial microtrauma and activation of the coagulation cascade [2]. Anatomical variations that act to narrow the thoracic outlet such as cervical ribs, fibromuscular bands, and hypertrophied musculature are well known to exacerbate the development of Paget-Schroetter syndrome [3]. Less frequent causative mechanisms such as posterior dislocations of the clavicular head [4], pseudoarthrosis of the clavicle [5], and Langer's axillary arch [6] have previously been reported as precipitating upper limb DVT. This is the first reported case of a sternoclavicular osteophyte secondary to osteoarthritis causing subclavian vein obstruction and upper limb deep venous thrombosis.

Degenerative osteoarthritis of the sternoclavicular joint is a relatively common condition, especially in the elderly population [7], with osteophytic projections being a frequent part of the pathological process [8]. It is seen more commonly in active people and extensive rowing experience of the patient presented in this case was likely a contributing factor to the development of his degenerative joint changes.

This anatomical variant presents diagnostic and therapeutic challenges. The presence and impact of the osteophytic projection could not be fully appreciated on initial presentation as extensive thrombus in the vasculature rendered visualisation of the course and calibre of the subclavian vein difficult on CT imaging. The significance of the osteophytic projection and the severe compression it imparted on the subclavian vein could only be appreciated after surgical intervention and placement of the radiolucent stent.

The management of this condition, once identified, is challenging. The accepted treatment for sternoclavicular arthrosis causing significant pain or functional impairment is surgical resection of the medial clavicular head, as less invasive treatment is unlikely to be effective [9, 10]. In an appropriate management plan in patients with symptomatic sternoclavicular arthrosis refractory to conservative management, this procedure imparts significant functional impairment and a substantial impact on quality of life [9]. In view of the patient's active lifestyle, clavicular head resection was not an appropriate intervention and the decision was made to continue the treatment conservatively with ongoing anticoagulation.

## 4. Conclusion

Paget-Schroetter syndrome is common in younger active patient groups. When occurring in more elderly patients other aetiologies such as abnormal anatomical compression of the subclavian vein should be considered. As seen in this case, degenerative osteoarthritis with osteophytic development can significantly compress posterior structures and may not be appreciated until the full course of the subclavian vein is possible to be visualized.
